# Supplementary material for: The Status of Wildlife Damage Compensation in China
Source: Animals (Basel). 2024 Jan 17;14(2):292. doi: 10.3390/ani14020292 (PMC10812642; doi:10.3390/ani14020292)
Supplement: Supplementary file 1 [file animals-14-00292-s001.zip › Supplementary material S1_revised.pdf]

Table S1: Species list of nuisance species, their IUCN conservation status, and the type of damage they cause, reported by 19 Chinese provinces, autonomous regions, and municipalities in the seven biogeographic regions of China.

Biogeographic region (Sun et al., 2020)	Provinces, Autonomous regions & Municipalities	Animal class	English name	Latin name	Conservation Status (IUCN)	Type of damage	References
**North (temperate grassland)**	Beijing	mammal	tolai hare	*Lepus tolai*	LC	crop damage	88
	Beijing	mammal	European badger	*Meles meles*	LC	crop damage	88
	Beijing	mammal	wild boar	*Sus scrofa*	LC	crop damage	88
	Beijing	mammal	leopard cat	*Prionailurus bengalensis*	LC	crop damage	31
	Beijing	mammal	rhesus macaque	*Macaca mulatta*	LC	crop damage	31
	Beijing	bird	Magpie	*Pica pica*	LC	crop, fruit damage	88
	Beijing	bird	Eurasian tree sparrow	*Passer montanus*	LC	crop, fruit damage	88
	Beijing	bird	Azure-winged magpie	*Cyanopica cyana*	LC	crop, fruit damage	88
	Beijing	bird	Eurasian eagle-owl	*Bubo bubo*	LC	crop, fruit, poultry damage	88
	Beijing	reptile	short-tailed pitviper	*Gloydius brevicaudus*	LC	human casualties	89
**North (temperate grassland)**	Tianjin	mammal	tolai hare	*Lepus tolai*	LC	crop damage	49
	Tianjin	mammal	Siberian chipmunk	*Tamias sibiricus*	LC	crop, fruit damage	49
	Tianjin	mammal	leopard cat	*Prionailurus bengalensis*	LC	poultry damage	49
	Tianjin	bird	Oriental white stork	*Ciconia boyciana*	EN	fish, shrimp damage	49
	Tianjin	bird	Great egret	*Ardea alba*	LC	fish, shrimp damage	49
	Tianjin	bird	Little egret	*Egretta garzetta*	LC	fish, shrimp damage	49
	Tianjin	bird	Grey crane	*Grus grus*	LC	fish, shrimp damage	49
	Tianjin	bird	White spoonbill	*Platalea leucorodia*	LC	fish, shrimp damage	49
	Tianjin	bird	Whooper swan	*Cygnus cygnus*	LC	fish, shrimp damage	49
	Tianjin	bird	Black-headed gull	*Chroicocephalus ridibundus*	LC	fish, shrimp damage	49
	Tianjin	bird	Cormorant	*Phalacrocorax carbo*	LC	fish, shrimp damage	49
	Tianjin	bird	Relict gull	*Ichthyaetus relictus*	VU	fish, shrimp damage	49
	Tianjin	bird	Mallard	*Anas platyrhynchos*	LC	fish, shrimp damage	49
	Tianjin	bird	Purple heron	*Ardea purpurea*	LC	fish, shrimp damage	49
	Tianjin	bird	Magpie	*Pica pica*	LC	crop, fruit damage	49
	Tianjin	bird	Eurasian tree sparrow	*Passer montanus*	LC	crop, fruit damage	49
**Northeast (coniferous forest)**	Heilongjiang	mammal	Siberian tiger	*Panthera tigris altaica*	EN	livestock damage	90
	Heilongjiang	mammal	Asiatic black bear	*Ursus thibetanus*	VU	human, livestock, fruit, apiculture damage	91
	Heilongjiang	mammal	wild boar	*Sus scrofa*	LC	crop, forest damage	92
	Heilongjiang	bird	Swan goose	*Anser cygnoides*	EN	crop damage	93
	Heilongjiang	bird	Taiga bean goose	*Anser fabalis*	LC	crop damage	54
	Heilongjiang	bird	Oriental white stork	*Ciconia boyciana*	EN	crop, fish damage	93
	Heilongjiang	bird	Grey crane	*Grus grus*	LC	crop, fish damage	93
	Heilongjiang	bird	Grey heron	*Ardea cinerea*	LC	crop, fish damage	93

	Heilongjiang	bird	Mallard	*Anas platyrhynchos*		crop, fish damage	93
**Northeast (coniferous forest)**	Jilin	mammal	Siberian tiger	*Panthera tigris altaica*	EN	livestock damage	33, 65
	Jilin	mammal	Amur leopard	*Panthera pardus orientalis*	VU	livestock damage	65, 94
	Jilin	mammal	Asiatic black bear	*Ursus thibetanus*	VU	human, apiculture damage	33, 65
	Jilin	mammal	Eurasian lynx	*Lynx lynx*	LC	livestock damage	65
	Jilin	mammal	leopard cat	*Prionailurus bengalensis*	LC	livestock damage	65
	Jilin	mammal	wolf	*Canis lupus*	LC	livestock damage	65
	Jilin	mammal	Siberian weasel	*Mustela sibirica*	LC	livestock damage	65
	Jilin	mammal	yellow-throated marten	*Martes flavigula*	LC	livestock damage	65
	Jilin	mammal	badger	*Meles meles*	LC	crop damage	65
	Jilin	mammal	sika deer	*Cervus nippon*	LC	fruit, seedling damage	95
	Jilin	mammal	Siberian roe deer	*Capreolus pygargus*	LC	fruit, seedling damage	95
	Jilin	mammal	red deer	*Cervus elaphus*	LC	crop damage	65
	Jilin	mammal	wild boar	*Sus scrofa*	LC	crop damage	33, 65, 95
	Jilin	bird	birds of prey	*Falconiformes /Strigiformes*	-	human, livestock damage	65
	Jilin	bird	geese and ducks	*Anseriformes*	-	crop damage	65
**Central (deciduous broad-leafed forest)**	Hunan	mammal	leopard cat	*Prionailurus bengalensis*	LC	Livestock damage	96
	Hunan	mammal	small Indian civet	*Viverricula indica*	LC	poultry damage	96
	Hunan	mammal	hog badger	*Arctonyx collaris*	VU	crop, livestock, poultry damage	96
	Hunan	mammal	Asiatic black bear	*Ursus thibetanus*	VU	crop damage	96
	Hunan	mammal	red-hipped squirrel	*Dremomys pyrrhomerus*	LC	crop damage	96
	Hunan	mammal	Chinese hare	*Lepus sinensis*	LC	crop damage	96
	Hunan	mammal	Tibetan macaque	*Macaca thibetana*	NT	crop, human casualties	97
	Hunan	mammal	rhesus macaque	*Macaca mulatta*	LC	crop, human casualties	96
	Hunan	mammal	tufted deer	*Elaphodus cephalophus*	NT	crop damage	96
	Hunan	mammal	Reeves's muntjac	*Muntiacus reevesi*	LC	crop damage	96
	Hunan	mammal	Chinese goral	*Naemorhedus griseus*	VU	crop damage	96
	Hunan	mammal	wild boar	*Sus scrofa*	LC	crop damage	96
	Hunan	mammal	Pere David's deer	*Elaphurus davidianus*	EW	crop damage	98
	Hunan	reptile	many-banded krait	*Bungarus multicinctus*	LC	human casualties	99
	Hunan	reptile	banded krait	*Bungarus fasciatus*	LC	human casualties	99
	Hunan	reptile	hundred-pace viper	*Deinagkistrodon acutus*	VU	human casualties	99
	Hunan	reptile	master viper	*Trimeresurus stejnegeri*	LC	human casualties	99
	Hunan	reptile	Chinese cobra	*Naja atra*	VU	human casualties	99
	Hunan	reptile	king cobra	*Ophiophagus hannah*	VU	human casualties	99
	Hainan	mammal	wild boar	*Sus scrofa*	LC	crop, poultry, property, human casualties	48
	Hainan	mammal	rhesus macaque	*Macaca mulatta*	LC	poultry, human casualties	48
	Hainan	mammal	Hainan Eld's deer	*Cervus eldii hainanus*	EN	crop, human casualties	48

South (tropical forest)	Hainan	mammal	red-hipped squirrel	*Dremomys pyrrhomerus*	LC	crop damage	48
	Hainan	mammal	red fox	*Vulpes vulpes*	LC	poultry damage	48
	Hainan	mammal	Yellow-bellied weasel	*Mustela kathiah*	LC	crop, poultry damage	48
	Hainan	mammal	leopard cat	*Prionailurus bengalensis*	LC	crop, poultry damage	48
	Hainan	mammal	vesper bat	*Vespertilionidae sp.*	-	fruit, crop damage	48
	Hainan	mammal	rat	*Muroidea sp.*	-	fruit, crop, poultry, human casualties	48
	Hainan	bird	Eurasian tree sparrow	*Passer montanus*	LC	crop damage	48
	Hainan	bird	Crested myna	*Acridotheres cristatellus*	LC	crop damage	48
	Hainan	bird	eagle species	*Aquila sp.*	-	fruit, poultry damage	48
	Hainan	bird	Little egret	*Egretta garzetta*	LC	crop, fish, shrimp damage	48
	Hainan	bird	Red-billed starling	*Sturnus sericeus*	LC	crop, fruit damage	48
	Hainan	bird	Whiskered tern	*Chlidonias hybrida*	LC	crop, fish, shrimp damage	48
	Hainan	bird	Turtle dove	*Streptopelia turtur*	VU	crop damage	48
	Hainan	bird	Chinese pond heron	*Ardeola bacchus*	LC	crop, fish, shrimp damage	48
	Hainan	bird	owl species	*Strigiformes sp.*	-	poultry	48
	Hainan	bird	Lesser coucal	*Centropus bengalensis*	LC	crop, fruit damage	48
	Hainan	bird	Chinese francolin	*Francolinus pintadeanus*	LC	crop damage	48
	Hainan	bird	Siberian crane	*Grus leucogeranus*	CR	crop, fish, shrimp	48
	Hainan	reptile	Burmese python	*Python bivittatus*	VU	poultry, livestock, human casualties	48
Southwest (alpine grassland)	Qinghai	mammal	Asiatic black bear	*Ursus thibetanus*	VU	livestock, human casualties	100
	Qinghai	mammal	brown bear	*Ursus arctos*	LC	livestock, human, house damage	101, 102
	Qinghai	mammal	wolf	*Canis lupus*	LC	livestock damage	100
	Qinghai	mammal	snow leopard	*Panthera uncia*	VU	livestock damage	100
	Qinghai	mammal	plateau pika	*Ochotona curzoniae*	LC	pasture damage	103
	Qinghai	mammal	wild yak	*Bos mutus*	VU	livestock, human, house damage	101
Southwest (alpine grassland)	Xizang	mammal	Himalayan marmots	*Marmota himalayana*	LC	pasture damage	56, 104
	Xizang	mammal	plateau pikas	*Ochotona curzoniae*	LC	pasture damage	56, 104,105
	Xizang	mammal	Tibetan macaque	*Macaca thibetana*	NT	crop damage	56
	Xizang	mammal	wolf	*Canis lupus*	LC	livestock damage	56,106, 107
	Xizang	mammal	red fox	*Vulpes vulpes*	LC	livestock damage	56
	Xizang	mammal	Eurasian lynx	*Lynx lynx*	LC	livestock damage	56
	Xizang	mammal	snow leopard	*Panthera uncia*	VU	livestock damage	56,106,107
	Xizang	mammal	Asiatic black bear	*Ursus thibetanus*	VU	house, livestock, crop damage	56,106,107
	Xizang	mammal	brown bear	*Ursus arctos*	LC	human, house, livestock, crop damage	56,106,107
	Xizang	mammal	white-lipped deer	*Przewalskium albirostris*	VU	crop damage	56,107

	Xizang	mammal	wild yak	*Bos mutus*	VU	livestock, human, house, pasture damage	56,106
	Xizang	mammal	kiang	*Equus kiang*	LC	pasture damage	56
	Xizang	mammal	bharal or blue sheep	*Pseudois nayaur*	LC	house, crop damage	106
**Northwest (desert)**	Xinjiang	mammal	dhole or red dog	*Cuon alpinus*	EN	livestock damage	37
	Xinjiang	mammal	desert hare	*Lepus tibetanus*	LC	crop damage, seedling damage	108
	Xinjiang	mammal	wolf	*Canis lupus*	LC	livestock, poultry damage	109
	Xinjiang	mammal	brown bear	*Ursus arctos*	LC	livestock, property damage	109
	Xinjiang	mammal	snow leopard	*Panthera uncia*	VU	livestock, property damage	109
	Xinjiang	mammal	wild boar	*Sus scrofa*	LC	crop, livestock, pasture, human casualties	109
**South (tropical forest)**	Guangxi	mammal	rhesus macaque	*Macaca mulatta*	LC	crop, human casualties	32
	Guangxi	mammal	wild boar	*Sus scrofa*	LC	crop, human casualties	110
	Guangxi	reptile	Burmese python	*Python bivittatus*	VU	livestock, poultry damage	111
**South (tropical & deciduous forest)**	Sichuan	mammal	Asiatic black bear	*Ursus thibetanus*	VU	crop, livestock damage	24,112
	Sichuan	mammal	leopard ssp.	*Panthera pardus* ssp.	VU	livestock damage	24,112
	Sichuan	mammal	leopard cat	*Prionailurus bengalensis*	LC	livestock, poultry damage	24,112
	Sichuan	mammal	civet cat	*Paguma larvata*	LC	crop, fruit damage	24,112
	Sichuan	mammal	Siberian weasel	*Mustela sibirica*	LC	Livestock & poultry damage, crop damage	24,112
	Sichuan	mammal	hog badger	*Arctonyx collaris*	VU	crop damage	24,112
	Sichuan	mammal	Tibetan macaque	*Macaca thibetana*	NT	crop damage	24,112
	Sichuan	mammal	Chinese porcupine	*Hystrix brachyura hodgsoni*	LC	crop damage	24,112
	Sichuan	mammal	Swinhoe's striped squirrel	*Tamiops swinhoei*	LC	crop damage	112
	Sichuan	mammal	tolai hare	*Lepus tolai*	LC	crop damage	112
	Sichuan	mammal	Sichuan takin	*Budorcas taxicolor tibetana*	VU	crop, forest damage	112
	Sichuan	mammal	northern red muntjac	*Muntiacus vaginalis*	LC	crop damage	112
	Sichuan	mammal	wild boar	*Sus scrofa*	LC	crop damage	24, 112
	Sichuan	mammal	wolf	*Canis lupus*	LC	livestock damage	24
	Sichuan	mammal	dhole or red dog	*Cuon alpinus*	EN	livestock damage	24
	Sichuan	mammal	South Asian sambar	*Rusa unicolor*	VU	crop damage	24
	Sichuan	mammal	giant panda	*Ailuropoda melanoleuca*	VU	crop damage	24
	Sichuan	bird	Jungle crow	*Corvus macrorthynchos*	LC	crop, fruit damage	112
**South (tropical forest)**	Yunnan	mammal	Asiatic black bear	*Ursus thibetanus*	VU	crop, property, human casualties	55
	Yunnan	mammal	wolf	*Canis lupus*	LC	livestock damage	113
	Yunnan	mammal	dhole or red dog	*Cuon alpinus*	EN	livestock damage	113
	Yunnan	mammal	rhesus macaque	*Macaca mulatta*	LC	crop damage	36
	Yunnan	mammal	South Asian sambar	*Rusa unicolor*	VU	crop damage	36
	Yunnan	mammal	Chinese porcupine	*Hystrix brachyura hodgsoni*	LC	crop damage	114

	Yunnan	mammal	bamboo rat	*Rhizomys sp.*	-	crop damage	114
	Yunnan	mammal	squirrel species	*Sciuridae sp.*	-	crop damage	114
	Yunnan	mammal	grey langur	*Trachypithecus phayrei*	EN	crop damage	114
	Yunnan	mammal	western black crested gibbon	*Nomascus concolor*	CR	crop damage	114
	Yunnan	mammal	Asian elephant	*Elephas maximus*	EN	crop, property, human casualties	55
	Yunnan	mammal	wild boar	*Sus scrofa*	LC	crop, property, human casualties	55
	Yunnan	mammal	gaur	*Bos gaurus*	VU	crop damage	55
	Yunnan	mammal	Indochinese leopard	*Panthera pardus delacouri*	VU	livestock and poultry damage	116
	Yunnan	bird	Grey-headed parakeet	*Psittacula finschii*	NT	crop damage	36
	Yunnan	bird	Eurasian tree sparrow	*Passer montanus*	LC	crop damage	114
**North (temperate grassland)**	Inner Mongolia	mammal	wolf	*Canis lupus*	LC	livestock, poultry damage	115
**Northwest (desert)**	Gansu	mammal	snow leopard	*Panthera uncia*	VU	livestock damage	34
	Gansu	mammal	wolf	*Canis lupus*	LC	livestock damage	34
	Gansu	mammal	Eurasian lynx	*Lynx lynx*	LC	livestock damage	34
	Gansu	mammal	brown bear	*Ursus arctos*	LC	livestock damage	34
**Central (deciduous broad-leafed forest)**	Shaanxi	mammal	Golden takin	*Budorcas taxicolor bedfordi*	VU	human casualties, property damage	33
	Shaanxi	mammal	black bear	*Ursus thibetanus*	VU	crop damage	33
	Shaanxi	mammal	Sumatran serow	*Capricornis sumatraensis*	Vu	crop damage	33
	Shaanxi	mammal	North China leopard	*Panthera pardus japonensis*	VU	livestock and poultry damage	33
	Shaanxi	mammal	rhesus macaque	*Macaca mulatta*	LC	crop damage	33
	Shaanxi	mammal	wild boar	*Sus scrofa*	LC	crop damage	33
	Shaanxi	mammal	tolai hare	*Lepus tolai*	LC	crop damage	33
	Shaanxi	mammal	Reeves's muntjac	*Muntiacus reevesi*	LC	crop damage	33
	Shaanxi	mammal	hog badger	*Arctonyx collaris*	VU	crop damage	33
	Shaanxi	bird	Crested ibis	*Nipponia nippon*	EN	crop damage	33
	Shaanxi	bird	Eagle owl	*Ninox scutulata*	LC	poultry damage	33
**Southeast (evergreen broad-leaved forest)**	Guizhou	mammal	black bear	*Ursus thibetanus*	VU	human casualties, crop damage	117
	Guizhou	mammal	wild boar	*Sus scrofa*	LC	human casualties, crop damage	117
	Guizhou	mammal	rhesus macaque	*Macaca mulatta*	LC	human casualties, crop damage, property damage	117
	Guizhou	mammal	leopard cat	*Prionailurus bengalensis*	LC	livestock damage	117
	Guizhou	mammal	Siberian weasel	*Mustela sibirica*	LC	poultry damage	117
	Guizhou	mammal	yellow-throated marten	*Martes flavigula*	LC	poultry damage	117

	Guizhou	mammal	dhole or red dog	*Cuon alpinus*	EN	livestock damage	117
	Guizhou	mammal	François' langur	*Trachypithecus francoisi*	EN	property damage	117
	Guizhou	mammal	Reeves's muntjac	*Muntiacus reevesi*	LC	crop damage	117
	Guizhou	bird	falcon species	*Falconidae spp.*	-	poultry damage	117
**East (evergreen broad-leaved forest)**	Anhui	mammal	wild boar	*Sus scrofa*	LC	human casualties, crop damage	118
**North (temperate grassland)**	Shanxi	mammal	North China leopard	*Panthera pardus japonensis*	VU	livestock damage	38, 119, 120
	Shanxi	mammal	tolai hare	*Lepus tolai*	LC	sapling	119,120
	Shanxi	mammal	wild boar	*Sus scrofa*	LC	crop damage, sapling	119, 120
	Shanxi	mammal	rhesus macaque	*Macaca mulatta*	LC	crop damage, human casualties，property damage	120
**Northeast (coniferous forest)**	Liaoning	bird	Vega gull	*Larus vegae*	LC	fish	57
