# Supplementary material for: The Status of Wildlife Damage Compensation in China
Source: Animals (Basel). 2024 Jan 17;14(2):292. doi: 10.3390/ani14020292 (PMC10812642; doi:10.3390/ani14020292)
Supplement: Supplementary file 1 [file animals-14-00292-s001.zip › Supplementary material S2.pdf]

Table S2. The standards of wildlife damage compensation management in 14 Chinese provinces or autonomous regions.

No.	Area	Year	Personal injury				Property damage		
			Personal injury causing no loss of labor force	Personal injury causing partial loss of labor force	Personal injury causing total loss of labor force	Personal injury causing death	Crops or economic forests	Livestock and poultry	Legal property
1	Yunnan	1998	Compensation for medical expenses and lost income (max. twice the average annual salary of employees in the previous year in the respective county or city).	Compensation for medical expenses and lump-sum disability benefits (max. 4 times the average salary of employees in the previous year in the respective county or city).	Compensation for medical expenses and lump-sum disability benefits (max. 8 times the average annual salary of employees in the previous year in the respective county or city).	Compensation and funeral expenses (in total 8 times the average annual salary of employees in the previous year in the respective county or city).	——	——	——
2	Shaanxi	2004	Compensatory payment (max. 3 times the income of local farmers in the previous year).	Compensatory payment (max. 10 times the income of local farmers in the previous year).	Compensatory payment (max. 15 times the income of local farmers in the previous year).	Compensatory payment - including funeral allowance (in total 20 times the income of local farmers in the previous year).	If >60% of the total yield is lost, compensation will be given according to the damaged yield considering 50% of the actual price.	Compensation is 20% of the value of the injured livestock. Compensation for death is 50% of the value of the lost livestock.	——
3	Jilin	2007	Compensation is 80% of the medical expenses and lost income (max. twice the average annual salary of employees in the previous year in the respective county, city or district).	Compensation for medical expenses and disability benefits (max. 5 times the average annual salary of employees in the previous year in the respective county, city or district).	Compensation for medical expenses and disability benefits (max. 10 times the average annual salary of employees in the previous year in the respective county, city or district).	Compensation for fatalities and funeral expenses (in total 10 times the average annual salary of employees in the previous year in the respective county, city or district).	Compensation is based on the average local yield per unit area of the damaged crop for the previous three years. 60% of the local market price of the damaged crop will be compensated.	Compensation for medical expenses of injured livestock but not more than 30% of the value of the injured livestock. Compensation for dead livestock is based on the actual value of the lost livestock.	——
4	Beijing	2009	——	——	——	——	Compensation for 60-80% of total loss.	Compensation is 50-70% of the medical expenses for injured livestock and poultry (max. 50% of the market price for poultry and livestock). Compensation for dead livestock and poultry is 60 -80% of the market price.	——
5	Gansu	2010	Compensation for medical expenses and lost income (max. 3 times the average income of local farmers in the previous year).	Compensation for the partial loss of labor force (max. 10 times or 15 times the average income of local farmers in the previous year).	Compensation for medical expenses and lump-sum disability benefits (max. 18 times the average income of local farmers in the previous year).	Compensation and funeral expenses (max. 20 times the average income of local farmers in the previous year).	Compensation based on the local market price.	Compensation for injured livestock max. 20% of the market price. Compensation for dead livestock is 80% of the market price.	Compensation is based on the local market price.

No.	Area	Year	Personal injury				Property damage		
			Personal injury causing no loss of labor force	Personal injury causing partial loss of labor force	Personal injury causing total loss of labor force	Personal injury causing death	Crops or economic forests	Livestock and poultry	Legal property
6	Tibet	2010	Compensation for medical expenses and lost income according to the per capita net income of local farmers and herdsmen in the previous year based on data from statistics department of the autonomous region.	Compensation for medical expenses and disability benefits (compensation is 15 times the average annual income of local farmers and herdsmen based on data from the statistics department of the autonomous region).	Compensation for medical expenses and disability benefits (compensation is 25 times the average annual income of local farmers and herdsmen based on data from the statistics department of the autonomous region)	Compensation for medical expenses, funeral expenses (1000 Yuan), and for fatalities is 30 times the average annual income of local farmers and herdsmen based on data from the statistics department of the autonomous region)	Compensation is 70% of the average annual market price of similar products in the previous year in the victim's county or city.	Compensation for dead livestock prices vary by animal species.	If repairable, compensation is 70% of the local average market price of the previous year's maintenance costs.<br>If not repairable, compensation is 50% of the local average market price of the previous year.
7	Qinghai	2011	Compensation for medical expenses and lost income based on the per capita net income of farmers and herdsmen of the province in the previous year, multiplied by the number of sick days.	Lump-sum disability benefits (max. 15 times the average annual net income of farmers and herdsmen of the province in the previous year).	Lump-sum disability benefits (max. 20 times the average annual net income of farmers and herdsmen of the province in the previous year).	Compensation for fatalities and funeral expenses (total amount is 22 times the average annual net income of farmers and herdsmen of the province in the previous year).	Compensation is 50% of the average local market price of the previous year.	Compensation is 50% of the average local market price.	Compensation is 50% of the average local market price.
8	Anhui	2019	Compensation is 80% of medical expenses and lost income based on the average daily income of employees in the previous year in the respective county or city.	Compensation for medical expenses (max. 4 times the average annual income of employees in the previous year in the respective county or city).	Compensation for medical expenses (max. 8 times the average annual income of employees in the previous year in the respective county or city).	Compensation for medical expenses and funeral expenses (in total 10 times the average annual income of employees in the previous year in the respective county or city).	Compensation is 60% of the total loss.	Compensation for injured livestock and poultry is 50-70% of the medical expenses (max. 50% of the market price of poultry and livestock). Compensation for dead livestock and poultry is 60-80% of the market price).	——
9	Guizhou	2021	Compensation for medical expenses and lost income based on the net per capita income of employees in the previous year of the respective county or city.	Disability benefits are 1-10 times the average annual income of employees	Disability benefits are 1-10 times the average annual income of employees in the province of the previous year.	Compensation for funerals is twice the disposable per capita income of urban residents of Guizhou in the previous year. Compensation for fatalities is 10 times the average annual salary of employees in urban units the province in the previous year.	Compensation for 50% of total losses	The compensation for injured livestock, poultry and special farmed animals is 20% of the market price of the previous year. The compensation for the dead livestock is 50% of the market price.	If repairable, compensation is 70% of the local county average market price of the previous year's maintenance costs. If not repairable, compensation is 50% of the local county average market price of the previous year.

No.	Area	Year	Personal injury				Property damage		
			Personal injury causing no loss of labor force	Personal injury causing partial loss of labor force	Personal injury causing total loss of labor force	Personal injury causing death	Crops or economic forests	Livestock and poultry	Legal property
10	Heilongjiang	2021	Compensation for medical expenses and lost income (based on the net per capita income of local employees in the previous year multiplied by the number of sick days. (max. compensation is the annual per capita disposable income of permanent residents of the province in the previous year).	Compensation for medical expenses and lump-sum disability benefits (max. 5 times the annual, disposable per capita income of permanent residents of the province in the previous year).	Compensation for medical expenses and lump-sum disability benefits (max. 10 times the annual, disposable per capita income of permanent residents of the province in the previous year).	Compensation for fatalities and funeral expenses. In total 15 times the annual, disposable per capita income of permanent residents of the province in the previous year).	Compensation is 50% of the average local market price of the previous year.	Compensation for injured livestock and poultry is 30% of the market price in the previous year. Compensation for dead livestock is 50% of the market price.	Standard compensation is not more than 50% of the market value of the lost property.
11	Shanxi	2023	Compensation for medical expenses and lost income.	Compensation for medical expenses and lump-sum disability benefits.		Compensation for medical expenses，fatalities, and funeral expenses.	The compensation shall be 60% of the average local market price at the time of the damage.	Compensation for injured livestock and poultry is 30-50% of the market price. Compensation for the dead livestock is 80% of the market price.	——
**12**	Inner Mongolia	2023	Compensation for medical expenses and lost income based on the net per capita income of local employees in the previous year multiplied by the number of sick days. (max. is 3 times the annual, disposable per capita income of permanent residents of the respective municipality in the previous year).	Compensation for medical expenses and lost income (max. one lump-sum disability benefit for personal injury), or for partial loss of labor force (max. 5-10 times the annual, disposable per capita income of permanent residents of the municipality in the previous year). Compensation for total loss of labor force is 10-15 times the annual per capita disposable income of permanent residents of the municipality in the previous year.		Compensation for medical expenses, lost income, and funeral expenses (max. 15 times the annual, disposable per capita income of permanent residents of the respective municipality in the previous year).	Compensation is 6% of the average local market price of the previous year.	Compensation for injured livestock and poultry is 30% of the average market price. Compensation for dead livestock is 60% of the average market price.	Compensation is max. 50% of the market value of the lost property.
13	Sichuan	2023	Compensation for medical expenses and lost income (max. the annual, disposable per capita income of permanent residents of the province in the	Compensation for medical expenses and lump-sum disability benefits (max. 8 times the annual, disposable per capita income of permanent residents of the province in the	Compensation for medical expenses and lump-sum disability benefits (max. 12 times of the annual, disposable per capita income of permanent residents of the	Compensation for medical expense, fatalities, and funeral expenses (max. 15 times the annual, disposable per capita income of permanent residents of the	Compensation is 50% of the average local market price at the time of the damage.	Compensation for dead livestock and poultry is 50% of the average market price.	Compensation is 50% of the repair and replacement cost.

No.	Area	Year	Personal injury				Property damage		
			Personal injury causing no loss of labor force	Personal injury causing partial loss of labor force	Personal injury causing total loss of labor force	Personal injury causing death	Crops or economic forests	Livestock and poultry	Legal property
			previous year).	previous year).	province in the previous year).	province in the previous year).			
14	Liaoning	2023	Compensation for medical expenses and lost income (max, the annual, disposable per capita income of permanent residents of the province in the previous year).	Compensation for medical expenses, lost income, and lump-sum disability benefits (for partial loss of labor force the max. benefit is 5 times the annual, disposable per capita income of local permanent residents; for total loss of labor force the benefit is 10 times the annual, disposable per capita income of local permanent residents).		Compensation for fatalities and funeral expenses (max. 15 times the annual, disposable per capita income of local permanent residents).	Compensation should be based on the damaged area (compensation is 50% of the average local market price of the previous year).	Compensation for injured livestock and poultry is 30% of the average market price of the previous year. Compensation for the dead livestock is 50% of the average market price of the previous year.	If repairable, compensation is 70% of the average market price of the previous year's maintenance costs in the respective county. If not repairable, compensation is 50% of the average market price of the previous year's maintenance costs in the respective county.
