# Supplementary material for: The Status of Wildlife Damage Compensation in China
Source: Animals (Basel). 2024 Jan 17;14(2):292. doi: 10.3390/ani14020292 (PMC10812642; doi:10.3390/ani14020292)
Supplement: Supplementary file 1 [file animals-14-00292-s001.zip › Supplementary material S3.pdf]

Chinese References (including Supplementary Material S1,2)

9. 何謦成, 吴兆录. 我国野生动物肇事的现状及其管理研究进展[J]. 四川动物, 2010, (1): 141-143.

11. 宋志勇，罗杰斯，刘婷娇.景洪市野生动物肇事及补偿情况调查研究[J]. 林业调查规划, 2019, 44(3): 125-129.

12. 汪家军.关于建立钱江源国家公园野生动物肇事补偿机制的路径探索[J]. 林业建设, 2021, (2): 9-11.

22. 王洪杰, 张希武, 岳仲明. 中华人民共和国野生动物保护法释义[M]. 中国民主法治出版社, 北京, 中国, 2016.

28. 苏凯文，任婕，黄元，杨洁，温亚利.自然保护地人兽冲突管理现状、挑战及建议[J]. 野生动物学报, 2022, 43 (1): 259-265.

29. 蒋志刚. 动物行为原理与物种保护方法[M]. 北京: 科学出版社, 2004, 327-339.

33. 蔡炳城, 李青文, 郭立新, 等.野生动物肇事损害补偿调查[J].野生动物, 2011, 32(4): 228-232.

46. 原艺，赵荣.我国野生动物致害补偿机制与野生动物肇事公众责任保险制度比较[J]. 世界林业研究, 2022, 第 35 卷(2): 123-128.

48. 王文霞，周冉. 海南省陆生野生动物致害调查报告. 中国林业科学研究院林业科技信息研究所，北京，中国，2022.

49. 王文霞.天津省陆生野生动物致害调查报告. 中国林业科学研究院林业科技信息研究所，北京，中国，2022.

50. 龙耀. 野生动物致损与经济补偿的诸多选项[J]. 改革, 2018, (2): 146-158

51. 刘渊. 野生动物致害的生态保护补偿法律制度研究[J]. 现代商贸工业, 2017,第 38 卷(29): 131-132.

52. 王小芮. 我国野生动物致害补偿制度研究[D]. 甘肃政法大学, 2021.

54. 王悦. 群鸟北归, 万只豆雁嫩江“加餐. https://heilongjiang.dbw.cn/system/2016/04/15/057178784.shtml

55. 谢文芳, 宋军平, 苏海萍, 岩丙, 王巧燕. 西双版纳国家级自然保护区野生动物肇事补偿现状及缓解对策[J]. 林业调查规划, 2020, 45(2): 182-186.

56. 梁曾飞，李志国，彭泰来，邢元军，胡觉，冯强.西藏野生动物肇事补偿实施现状、存在问题以及对策[J].中南林业调查规划, 2020, 39(3): 36-41.

57. 岳巍. 鸟！别吃鱼~唉~吃吧. https://news.sina.com.cn/o/2008-03-30/071013656676s.shtml
64. 甘燕君，李玲.西双版纳州野生动物肇事补偿现状及补偿机制初探[J]. 绿色科技, 2018, (12): 38-41.
65. 孙丽，李伟东，艾平，郑振河，马琼芳，吴景才，孔维尧.吉林省野生动物损害特点及补偿工作评价[J]. 野生动物学报, 2015, (1): 48-53.
66. 周鸿升，唐景全，郭保香，王希群，董金花，李凯，侯森林.我国重点保护野生动物肇事问题、特点及其解决途径[J].北京林业大学学报·社会科学版, 2010, (2): 37-41.
67. 李雯雯.西双版纳人象冲突风险评估研究[D]. 云南财经大学, 2017.
72. 王一晴，戚新悦，高煜芳.人与野生动物冲突: 人与自然共生的挑战[J].科学, 2019, 第71卷(5): 1-4, 69-70.
73. 云南省人民政府. 1988. 云南省重点保护陆生野生动物造成人身财产损害补偿办法. https://www.yn.gov.cn/zwgk/zfxxgkpt/gkptzcwj/gz/202112/t20211222_231977.html.20230808 (1998.9.19)
74. 陕西省人民政府. 2004. 陕西省重点保护陆生野生动物造成人身财产损害补偿办法. http://www.shaanxi.gov.cn/zfxxgk/fdzdgknr/zcwj/nszfgz/202208/t20220804_2233088.html (2004.11.4)
75. 吉林省人民政府. 2007. 吉林省重点保护陆生野生动物造成人身财产损害补偿办法. http://xxgk.jl.gov.cn/szf/gkml/201812/t20181205_5349567.html
76. 北京市人民政府. 2009. 北京市重点保护陆生野生动物造成损失补偿办法. https://www.beijing.gov.cn/zhengce/zhengcefagui/201905/t20190529_82101.html
77. 甘肃省人民政府. 2010. 甘肃省陆生野生保护动物造成人身伤害和财产损失补偿办法. http://www.gansu.gov.cn/art/c103795/c104045/c104050/201011/206342.shtml
78. 西藏自治区人民政府. 2010. 西藏自治区陆生野生动物造成公民人身伤害或者财产损失补偿办法. http://www.xizang.gov.cn/zwgk/xxfb/fgwj/201902/t20190223_61885.html
79. 青海省人民政府. 2012. 青海省重点保护陆生野生动物造成人身财产损失补偿办法. http://sft.qinghai.gov.cn/pub/qhsfxzw/qhszfgzk/szfgz/202102/t20210203_56033.html
80. 安徽省人民政府. 2011. 安徽省陆生野生动物造成人身伤害和财产损失补偿办法. https://www.ah.gov.cn/public/1681/554081141.html

81. 贵州省林业局.贵州省陆生野生动物造成人身财产损害补偿办法. http://lyj.guizhou.gov.cn/zfxxgk/fdzdgknr/zcwj_5620825/ywwj_5620827/202109/t20210914_70393501.html
82. 黑龙江省人民政府. 2021. 黑龙江省陆生野生动物造成人身伤害和财产损失补偿办法. https://www.hlj.gov.cn/hlj/c107910/202112/c00_30633877.shtml
83. 山西省人民政府. 2023. 山西省陆生野生动物造成人身与财产损害补偿办法. http://www.shanxi.gov.cn/zfxxgk/zfxxgkzl/fdzdgknr/lzyj/szfl/202302/t20230203_7914604_gzk.shtml
84. 内蒙古自治区人民政府. 2023. 内蒙古自治区陆生野生动物致害补偿管理办法. https://www.nmg.gov.cn/zwgk/zfxxgk/zfxxgkml/gzxzgfxwj/xzgfxwj/202301/t20230119_2217604.html?dzb=true
85. 四川省人民政府. 2023. 四川省陆生野生动物致害补偿办法.
86. 辽宁省人民政府. 2023. 辽宁省陆生野生动物造成人身伤害和财产损失补偿办法. https://www.ln.gov.cn/web/zwgkx/zfwj/szfbgtwj/2023n/2023031408431910540/index.shtml (2023.2.23)
88. 李建. 北京市密云区野生动物肇事及补偿情况分析[J]. 绿化与生活, 2015, (12): 15-19.
89. 王方辰.当心被毒蛇咬伤－北京毒蛇伤人事件逐年上升[J]. 科技潮, 2001, (8): 35-37.
90. 侯鸣, 王松, 张涛, 孙晓宇, 董宝森. 黑龙江野生东北虎进村追踪:不到半年出现数次，“大王”为何频频“下山”？ http://www.xinhuanet.com/2021-04/24/c_1127370571.htm
91. 雪灵谷. 黑熊下山，在大街上狂奔，跑进一个小胡同后，就消失在茫茫夜色中. https://www.163.com/dy/article/HHL7CM3A0524I0P1.html
92. 梁书斌. 黑龙江村民遭野猪袭击受伤. http://news.sina.com.cn/c/2014-11-15/110831149269.shtml
93. 于淑鸿, 姚爱群, 吴德士. 五万只北飞大雁黑龙江畔歇脚. https://heihe.dbw.cn/system/2020/04/15/058394959.shtml.
94. 吉林省林业宣传中心. 珲春一只野生少年东北豹进入村院造成 30 多只鸡死亡，一只狗受伤. http://www.isenlin.cn/sf_65986208CF2C46519EE9B02BB7549EA0_209_4AC6F4AD498.html (2018.9.28)
95. 李祎斌，陈楚，刘丙万.吉林省珲春地区人与野生动物冲突现状与防控调查[J]. 野生动物学报, 2018, 第 39 卷(4): 962-965.

96. 刘美斯，廖庆义，白林壮，田书荣. 壶瓶山国家级自然保护区野生动物肇事现状及管理建议[J]. 野生动物学报, 2018, 39(1): 177-180.
98. 黄兴华. 湖南洞庭湖麋鹿迷途与人争食 菜农受损爱恨交加. http://news.sohu.com/20121024/n355587194.shtml
99. 段涵敏, 梁辉. 湖南暴雨逼毒蛇出洞, 至少 8 人被咬伤. http://hunan.ifeng.com/news/fghx/detail_2013_05/17/809594_0.shtml
https://www.sc.gov.cn/10462/zfwjts/2023/1/18/8152f96b3e4c4295b7ffdbc77b261dc8.shtml
100. 万玛加. 青海拟定陆生野生动物"肇事"赔偿试点方案. https://www.360kuai.com/pc/9be037ec463da3f98?cota=3&kuai_so=1&sign=360_57c3bbd1&refer_scene=so_1
101. 赵凛松. 青海实施野生动物伤人赔偿办法. https://www.chinanews.com/sh/2014/03-17/5960733.shtml
102. 范茹歌. 德令哈市森林公安局处置野生动物肇事事件. https://www.sohu.com/a/429809176_120207624
103. 汤中和，郭占泽，郭占显，郭万春.青海玉树市高原鼠兔危害情况调查[J].四川畜牧兽医, 2022, 49(12): 20-21, 24.
104. 魏学红，杨富裕，孙磊.高原鼠兔对西藏高寒草地的危害及防治[J].四川草原, 2006, (5): 41-42, 45.
105. 王永顺.高原鼠兔的危害现状调查与防治建议[J].兽医导刊, 2020, (18): 56.
106. 新华社. 西藏野生动物"肇事"政府补偿. http://news.sina.com.cn/s/2005-09-07/09416882374s.shtml
107. 刘洪明. 西藏 9 年间落实野生动物肇事补偿资金 4 亿多元. http://news.sina.com.cn/c/2015-02-15/155031524552.shtml
108. 张雷，杨树赛，王成. 新疆福海县野兔泛滥成灾,部分庄稼被毁. http://news.sina.com.cn/c/2008-06-11/083615721450.shtml
109. 新疆生产建设兵团林业局. 新疆生产建设兵团开展重点保护野生动物造成职工财产损害专项补助. http://finance.sina.com.cn/nongye/nygd/20150205/091921482453.shtml
110. 赵敏. 南宁 54 岁村妇被野猪突然袭击，全身多处被严重咬伤. https://baijiahao.baidu.com/s?id=1619171270459265100
111. 新华网. 广西：村民放羊被大蟒蛇追逐 山羊被活活缠死. http://news.sohu.com/20140923/n404567802.shtml

112. 谌利民, 熊跃武, 马曲波, 等. 四川唐家河自然保护区周边林缘社区野生动物冲突与管理对策研究[J].四川动物, 2006, 25(4):781-783.
114. 秦蒙琳, 史效轩. 云南野生动物肇事 5 年损失超 2.4 亿. https://www.kunming.cn/news/c/2013-08-29/3387295.shtml
115. 刘俊霞. 内蒙古草原生态环保面临新难题：野生动物致害 损失谁来补. http://inews.nmgnews.com.cn/system/2013/01/05/010894354.shtml
116. 李永祥.南滚河自然保护区野生动物肇事理赔现状调查[J]. 云南林业, 2020, (1): 80-85.
117. 彭典,陈鹏. 做好防范！贵州常见这些野生动物肇事. https://www.360kuai.com/pc/940e834b49b265ae6?cota=3&kuai_so=1&tj_url=so_vip&sign=360_57c3bbd1&refer_scene=so_1
119. 新华网. 山西部分山区时发野生动物害农事件. http://news.sohu.com/84/00/news207910084.shtml
120. 张建军.野生动物对群众生产生活造成损害情况的研究——以山西阳城蟒河猕猴国家级自然保护区为例[J].中国林业经济, 2019, (3): 120-123.
122. 王斌, 李雯雯, 许利剑, 陆英杰, 郭敏. 西双版纳自然保护区勐养子保护区亚洲象肇事特点及损失情况分析[J].林业调查规划, 2017, 42(1): 118-123, 129.
123. 郭贤明,王兰新. 西双版纳亚洲象食物源基地建设的思考[J]. 林业建设. 2019,6,30-33.
124. 达瓦次仁，弓进梅，拉巴卓嘎.改革开放以来西藏自然保护区建设与成就[J].西藏研究, 2018, (5): 133-140.
125. 人民日报. 吉林森林覆盖率提高到 45.2%. https://finance.sina.cn/2023-02-08/detail-imyeyaiy3164690.d.html
126. 甄春延.善待人类的朋友: 吉林省保护野生动物工作综述[J].吉林人大, 2013, (1): 5-7.
128. 侯一蕾,温亚利.野生动物肇事对社区农户的影响及补偿问题分析——以秦岭自然保护区群为例[J]. 林业经济问题, 2012, 32(5): 388-391.
130. 唐勤.西双版纳人象冲突与缓解对策[D]. 昆明理工大学, 2007
131. 云南省林业和草原局. 云南野生动物公众责任保险试点工作成效显著. http://lcj.yn.gov.cn/html/2012/mainnews_1031/30552.html
